# The Ash as a Remedy in Rheumatism and Gout

**Published:** 1852-12

**Authors:** 


Translated from the French—L'Union De La Louisiana

*The Ash as a Remedy in Rheumatism and Gout*, *by* De. Larue, D. M. P.

The Therapeutic agents for these diseases, which are found in the authors, are far from being satisfactory. For this reason, I propose to speak in detail, of a plant very generally spread through the temperate climates of Europe—the common Ash, (Fraxinus Excelsior) belonging to the Jesmine class of Jussieu.

In 1840 my mother, who had been more or less afflicted for two years, found herself the victim of a permanent and almost general chronic rheumatic gout. To her *critical* age—her sanguine-lymphatic temperament—a good and strong constitution—she added an hereditary influence. The pain was sharp, the tumefaction considerable, the movements were executed with difficulty, and, at times, were impossible. There was little or no fever, although, for the most part, the functions commenced to be sensibly altered. All the proper attempts to cure her having proved completely fruitless, she was about to discharge her physicians and give herself up to nature and time, when her seamstress urged her to use a ptisan of the leaves of the ash. Seeing no danger to arrive from it, I recommended her to try it. At the end of a fortnight she was much better, and at the end of a few months she was perfectly cured. Since that time she has continued to use it whenever she has apprehended or felt the return of the disease. Since this cure, so remarkable and dear to my heart, I have, many times, in every species of gout and rheumatism (acute, chronic, fixed, shifting, vague, or determined) prescribed the same remedy with entire success. In my experience I have used the leaves only. Gathered towards the end of June and dried, I have always ordered: 1st, a decoction of 10 to 12 grammes to 200 grammes of water, to be taken by the cupful every three hours, or only morning and evening, according to the intensity of the affection—2d, small lavements, to the number of two or three a day, of the same strength—3d, applied for some hours on the painful parts, sometimes over the whole body, after being previously heated.

*Reflections.*—Cesalpin, Lobel, Helwing, and Coste speak of the bark of the Ash as being a febrifuge, equal to Quinine, whilst others accord to the leaves a purgative or an astringent effect; and even, according to Gilbert, applicable to the treatment of scrofula. But none, to my knowledge, have attributed to it the virtues which my experiments would claim for it. Thus, without repelling the teachings of our predecessors, which, however, we believe are tainted with error or exaggeration, we prove that this substance may be administered for a long time without inconvenience, and has a general action so prompt as to apparently act specifically upon these diseases.

